# Differential gene expression patterns in Niemann-Pick Type C and Tay-Sachs diseases: Implications for neurodegenerative mechanisms

**DOI:** 10.1371/journal.pone.0319401

**Published:** 2025-03-19

**Authors:** Ramin Yousefpour Shahrivar, Fatemeh Karami, Ebrahim Karami

**Affiliations:** 1 School of Biological Sciences, Georgia Institute of Technology, Atlanta, Georgia, United States of America; 2 Department of Medical Genetics, Applied Biophotonics Research Center, Science and Research Branch, Islamic Azad University, Tehran, Iran; 3 Department of Electrical and Computer Engineering, Faculty of Engineering and Applied Sciences, Memorial University of Newfoundland, St. John’s, Canada; I3S: Universidade do Porto Instituto de Investigacao e Inovacao em Saude, PORTUGAL

## Abstract

Lysosomal storage disorders (LSDs) are a group of rare genetic conditions characterized by the impaired function of enzymes responsible for lipid digestion. Among these LSDs, Tay-Sachs disease (TSD) and Niemann-Pick type C (NPC) may share a common gene expression profile. In this study, we conducted a bioinformatics analysis to explore the gene expression profile overlap between TSD and NPC. Analyses were performed on RNA-seq datasets for both TSD and NPC from the Gene Expression Omnibus (GEO) database. Datasets were subjected to differential gene expression analysis utilizing the DESeq2 package in the R programming language. A total of 147 differentially expressed genes (DEG) were found to be shared between the TSD and NPC datasets. Enrichment analysis was then performed on the DEGs. We found that the common DEGs are predominantly associated with processes such as cell adhesion mediated by integrin, cell-substrate adhesion, and urogenital system development. Furthermore, construction of protein-protein interaction (PPI) networks using the Cytoscape software led to the identification of four hub genes: *APOE*, *CD44*, *SNCA*, and *ITGB5*. Those hub genes not only can unravel the pathogenesis of related neurologic diseases with common impaired pathways, but also may pave the way towards targeted gene therapy of LSDs.In addition, they serve as the potential biomarkers for related neurodegenerative diseases warranting further investigations.

## Introduction

**Fig 1:**
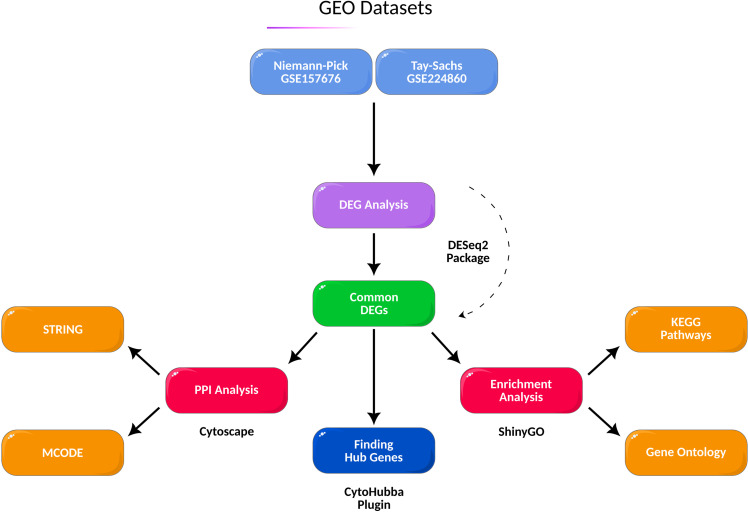
An overview of all the performed analyses.

Tay-Sachs Disease (TSD, MIM#272800) is classified as a rare autosomal recessive condition that predominantly impacts the neurological system and has a prevalence of about 1 in 320,000 live births in the general population [1]. It is caused by the genetic mutations within the *HEXA* gene, responsible for encoding the enzyme hexosaminidase A (Hex-A), which is involved in the degradation of GM2 ganglioside in neurons. Hex-A deficiency, therefore, causes the accumulation of GM2 ganglioside in nerve cells, leading to progressive damage in the nervous system [2]. Niemann-Pick Type C (NPC, MIM#257220) is a genetic disorder affecting lipid metabolism, particularly the metabolism of cholesterol, with an estimated prevalence of 1 in 150,000 live births [3]. Two studies estimated the prevalence of NPC in the United States and Quebec as 0.95 per million and 0.61 per 100,000 births, respectively [4, 5]. It is called childhood alzheimer and has different age onset and clinical manifestations. The primary pathogenesis of Niemann-Pick Disease (NPC) involves the disruption of lipid transport within cells, which is caused by mutations in either the *NPC1* or *NPC2* genes, with approximately 95% of cases associated with NPC1 [6, 7]. On the other hand, the buildup of GM2 ganglioside lipids in the brain is a consequence of mutations in the *HEXA* gene, resulting in TSD. TSD and NPC share common features in the accumulation of lipids and glycolipids and subsequent neurodegeneration and cognitive and motor deficits in affected individuals. In terms of the effects of TSD and NPC on cognition, different forms of psychosis were identified in TSD and NPC patients [8–10]. Previous studies have identified connections among some types of LSDs, such as Gaucher’s disease, NPC, Krabbe disease, Fabry disease, and Sandhoff disease, and various neurodegenerative diseases [11–16]. For example, research has shown that individuals diagnosed with Gaucher’s disease have a twenty-fold higher likelihood of developing Parkinson’s disease (PD) throughout the course of their lifetime [17]. A hypothesis was presented in 2014 suggesting that the presence of heterozygous mutations in the NPC gene might potentially serve as an autonomous risk factor for the development of Alzheimer’s Disease (AD) [11]. Moreover, in the event that asymptomatic individuals carrying *NPC1/2* mutations will be affected by AD, it is postulated that the use of miglustat, which is often suggested for the treatment of NPC and Gaucher disease type 1, may be a viable approach to consider [11]. Another piece of research found that the frequency of *NPC1* and *NPC2* variants was higher in patients with amyloid beta (Aβ) deposition, suggesting that variants in NPC genes may play a role as risk or disease-modifying factors for AD [18]. In this regard, finding common gene expression profiles may shed light on the pathogenesis of other neurodegenerative disorders and provide novel biomarkers to identify them in early stages. To elucidate the common involved genes in the neurodegenerative evolution of TSD and NPC, herein, it was aimed to explore the potential overlapping key genes, pathway networks, and gene ontologies related to the neural pathogenesis of NPC and TSD through various bioinformatics analyses (Fig 1).

## Materials and methods

### Data collection

Two recently published datasets, GSE224860 [19] and GSE157676 [20], for TSD and NPC, respectively, were obtained from the Gene Expression Omnibus (GEO) database [21] (Table 1).

**Table 1:** Detailed description of the used datasets.

Disease	TSD	NPC
**Platform**	GPL24676 Illumina NovaSeq 6000 (Homo sapiens)	GPL18573 Illumina NextSeq 500 (Homo sapiens)
**GEO ID**	GSE224860	GSE157676
**Control**	10	3
**Disease**	9	3

Both datasets contained RNA-seq count matrices for the healthy and patient groups. More specifically, two TSD fetal brain samples were compared against two control fetal brain samples. Both the TSD and NPC datasets were from the 17th gestational week.

### Quality control and normalization

**Fig 2:**
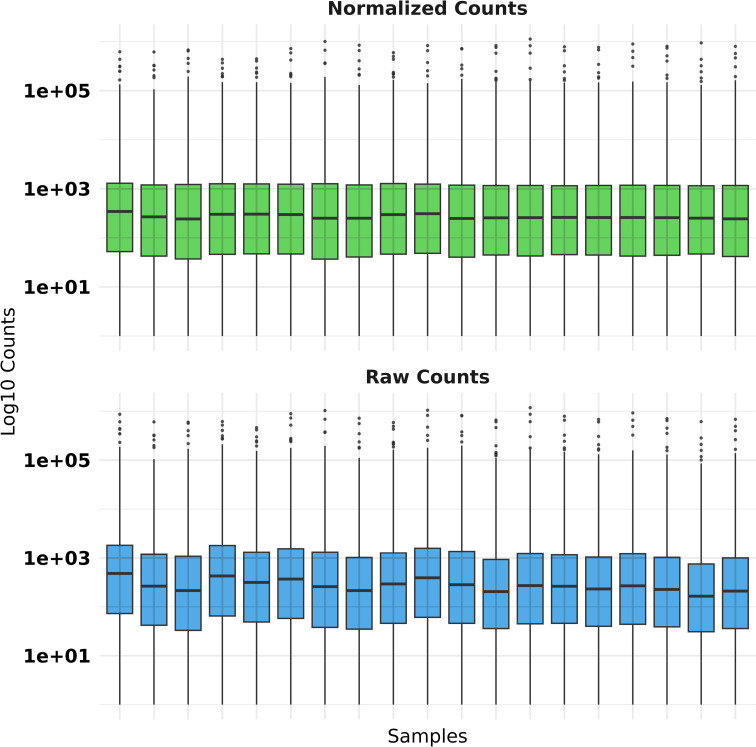
Quality control boxplot for TSD RNA-seq raw versus normalized counts using the DESeq2 package.

**Fig 3:**
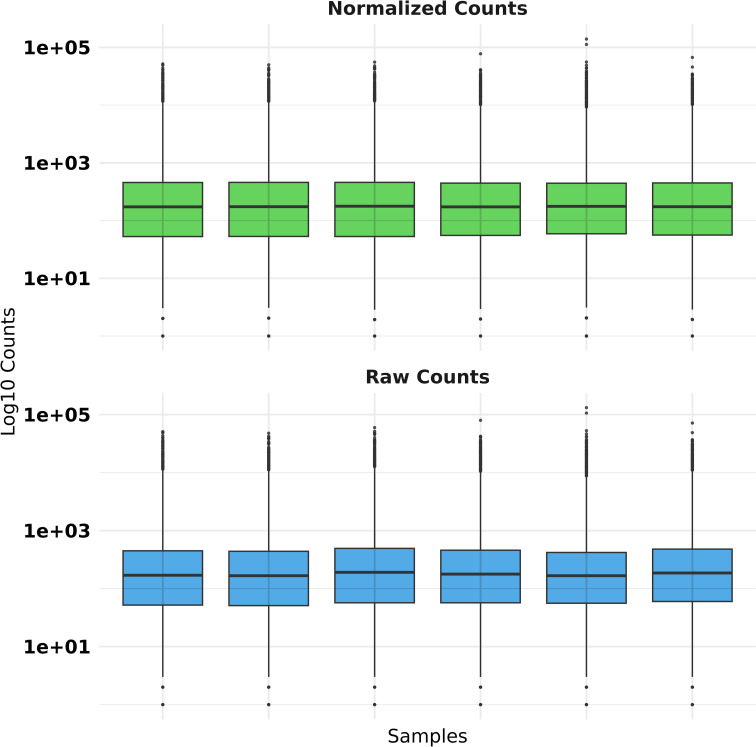
Quality control boxplot for NPC RNA-seq raw versus normalized counts using the DESeq2 package.

To ensure robust quality control of RNA-seq data, we filtered genes to retain only those with a minimum count of 10 in at least the smallest group of samples by removing low-expression genes likely to contribute noise rather than biological signal. We used the DESeq2 package to normalize the data to account for differences in library sizes across samples [22]. For visualization purposes, we added a pseudo count (+1 ) to avoid undefined values during log10 transformation and compared raw and normalized counts using faceted boxplots (Figs 2 and 3).

### Identification of DEGs

**Fig 4:**
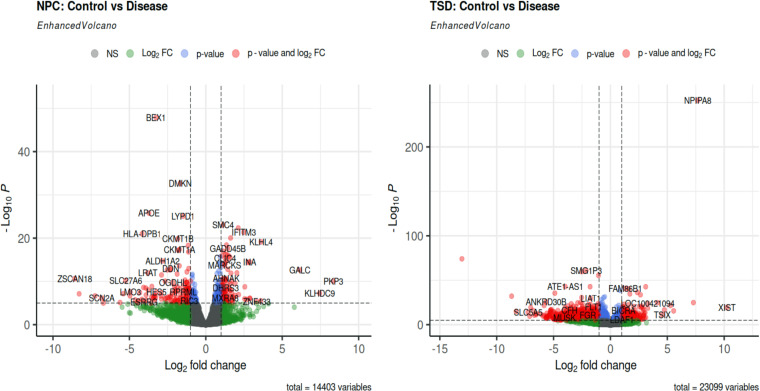
Volcano plots for TSD and NPC datasets. The volcano plot shows the spread of differentially expressed genes based on their LFC and padj values.

**Fig. 5:**
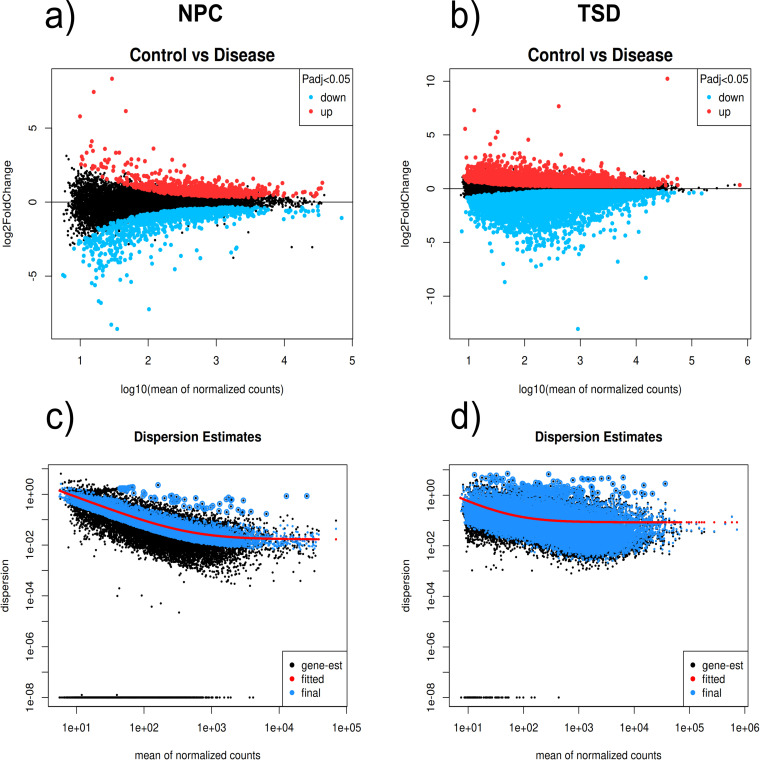
(a, b) MD plots for NPC and TSD, and (c, d) dispersion plots for NPC and TSD. In the MD plots, the vertical axis (y-axis) represents the log fold-change, where positive values indicate upregulated genes and negative values indicate downregulated genes in one condition. The horizontal axis (x-axis) represents the average gene expression across the two conditions, normalized and log10-transformed. In the dispersion plots, the x-axis shows the mean of normalized reads, and the y-axis represents the dispersion estimates. A well-fitted trend line is observed in both conditions.

Differential gene expression analysis was performed on each dataset separately using the DESeq2 package of the R programming language (R version 4.3.2, http://www.r-project.org/). Data pre-processing was conducted to exclude any gene that had a cumulative count of less than 10 across all samples. DEG identification was based on the criterion of having an absolute LFC>1 and a padj<0.05, using the Benjamini-Hochberg correction method. The ggplot2 package was used for the generation of volcano plots in this study (https://ggplot2.tidyverse.org/) (Fig 4). The mean-difference and dispersion plots were generated after running DESeq2 (Fig 5).

### Pathway and enrichment analyses

Gene Ontology (GO) enrichment and Kyoto Encyclopedia of Genes and Genomes (KEGG) pathways were identified and visualized using the ShinyGO web-based tool (version 0.77, http://bioinformatics.sdstate.edu/go/). The results were filtered using to keep the hits with a pvalue<0.05.

### PPI network and hub gene identification

A PPI network for overlapping DEGs was constructed using the STRING web-based tool (https://string-db.org/). The results of STRING were exported and visualized using Cytoscape (V3.10.1). The MCODE plugin (V2.0.3) [23] was used for the identification of significant gene modules in the DEG network. Furthermore, the CytoHubba plugin (V0.1) inside the Cytoscape software was used for the identification of hub genes [24]. Following evaluation of the hub genes, intersection of six different algorithms (MCC, MNC, Degree, Closeness, Radiality, and Stress) was used for the final result.

## Results

### DEGs identification

**Fig 6:**
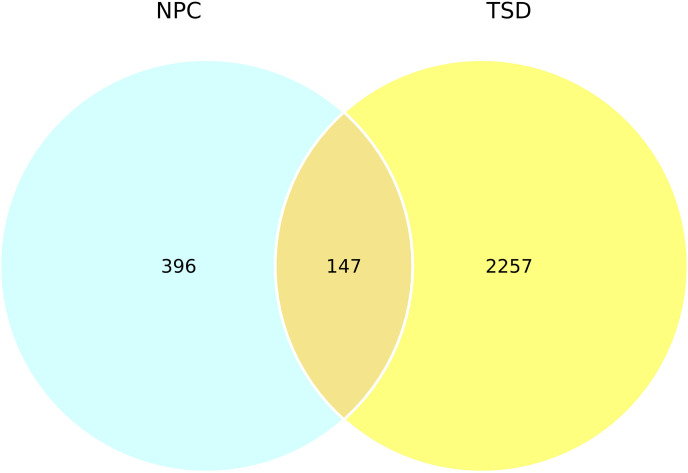
Venn diagram showing the overlap between TSD and NPC. A total of 147 DEGs were found to be shared. The diagram was generated using ggplot2.

A total of 2404 and 543 protein-coding DEGs were identified in the TSD and NPC datasets, respectively. Fig 4 displays the volcano plots for the NPC and TSD. A total of 147 protein-coding DEGs (a combination of both upregulated and downregulated genes) were found to be shared between the two datasets (Table 2), which is shown by the Venn diagram generated using R (Fig 6).

**Table 2:** Top 15 most downregulated genes in ascending order among 147 common deferentially expressed genes (DEGs), based on log2 fold change, p-value, and adjusted p-value (padj). A minus sign (-) indicates downregulated genes.

Gene Symbol	TSD (log2foldchange)	TSD (pvalue)	TSD (padj)	NPC (log2foldchange)	NPC (pvalue)	NPC (padj)
SPOCK3	−3.582	9.121e-15	1.177e-12	−8.289	7.436e-08	6.033e-06
ALDH1A2	−5.257	5.332e-17	1.257e-14	−2.837	1.892e-15	9.891e-13
ZIC1	−4.558	2.341e-12	1.369e-10	−3.522	3.925e-05	1.043e-03
EDNRB	−4.061	5.450e-14	5.403e-12	−3.656	3.287e-04	5.273e-03
TAC1	−3.544	3.580e-07	4.280e-06	−4.112	7.521e-05	1.715e-03
ESRRG	−3.254	5.968e-09	1.294e-07	−4.044	5.574e-06	2.223e-04
SLC27A6	−1.775	1.206e-05	8.524e-05	−5.248	6.025e-11	1.350e-08
AARD	−3.671	2.409e-10	8.018e-09	−2.609	3.740e-03	3.192e-02
PLA2G4A	−2.490	1.290e-11	6.070e-10	−3.780	9.136e-09	1.094e-06
WIF1	−3.108	1.146e-04	5.713e-04	−3.156	7.426e-04	9.895e-03
SLC24A4	−4.572	5.938e-11	2.282e-09	−1.687	5.193e-03	3.998e-02
LRAT	−2.414	3.324e-12	1.855e-10	−3.800	1.068e-12	3.313e-10
APOE	−2.464	1.559e-05	1.054e-04	−3.727	1.564e-26	7.360e-23
EFEMP1	−4.348	3.074e-07	3.755e-06	−1.734	5.162e-04	7.536e-03
IRS4	−4.622	1.148e-16	2.368e-14	−1.320	1.557e-06	7.631e-05

### Gene ontology and pathway analysis

**Fig 7:**
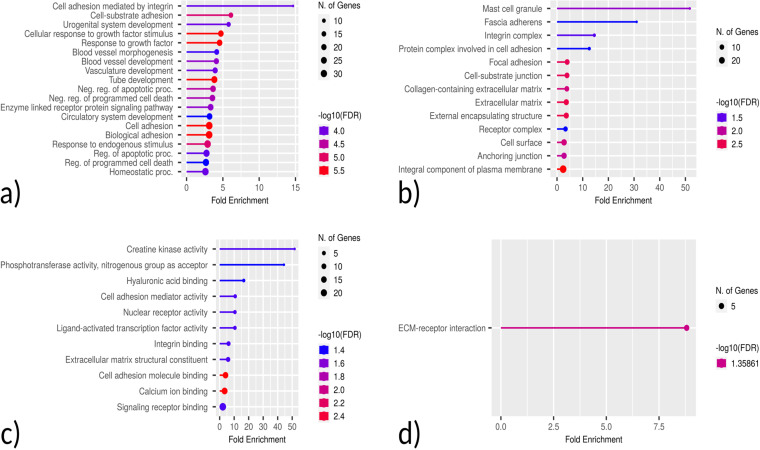
Enriched GO terms for the identified DEGs for TSD and NPC. a) Biological Process b) Cellular Component c) Molecular Function d) KEGG Pathways.

Based on GO enrichment, the biological process acted primarily on cell adhesion mediated by integrin and cell-substrate adhesion. These proteins were primarily located in the mast cell granule, fascia adherence, integrin complex, and protein complex involved in cell adhesion. With regard to molecular functions, the proteins played roles in creatine kinase activity and phosphotransferase activity (nitrogenous group as acceptor). According to KEGG pathway analysis, these proteins were primarily involved in the ECM-receptor interaction (Fig 7).

### PPI network and hub gene identification

**Fig 8:**
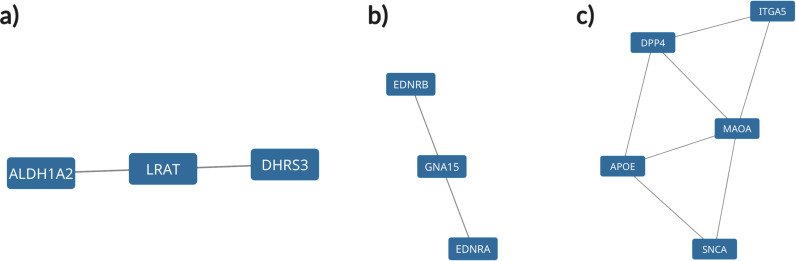
Significant clusters within the DEG network using the MCODE plugin in Cytoscape. Three cluster were identified with the MCODE 3. The third cluster (**c**) was found to be the most significant cluster.

**Fig 9:**
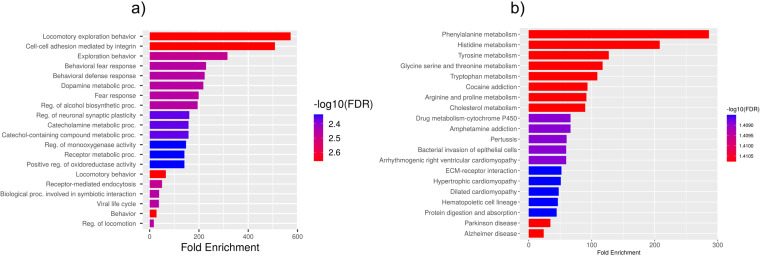
Enriched KEGG pathways for the highest scoring MCODE cluster within the constructed DEG network. Most of the top pathways are related to the metabolism of amino acids. a) GO-BP b) KEGG pathways.

**Fig 10:**
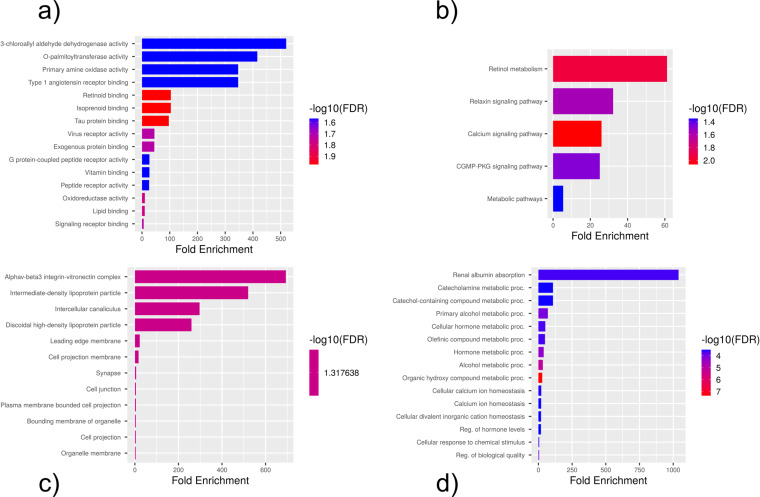
GO and KEGG pathway analyses for the combined MCODE clusters. a) GO-MF b) KEGG c) GO-CC d) GO-BP.

The PPI network for the 147 DEGs was constructed after the common DEGs were imported to STRING. The Molecular Complex Detection (MCODE) V1.5.1 plugin was used to identify significant clusters within the network. Three clusters were identified by MCODE; the most significant cluster had a 3.5 score, which included *SNCA*, *APOE*, *MAOA*, *DPP4*, and *ITGA5* genes (Fig 8-c). GO-BP analysis showed that this cluster is mostly involved in the locomotor exploration behavior. KEGG analysis showed that this cluster was enriched in amino acid metabolism pathways: phenylalanine, histidine, tyrosine, glycine, serine, threonine, and tryptophan metabolism (Fig 9). KEGG and GO analyses were also done for the clusters combined together, which showed retinol metabolism as the most enriched KEGG pathway (Fig 10).

**Fig 11:**
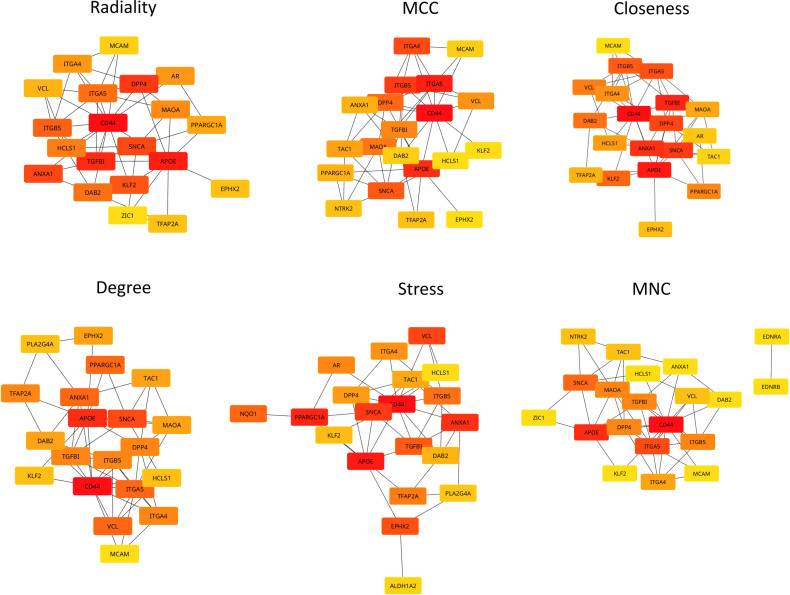
Identification of hub genes with six different algorithms using the CytoHubba plugin in Cytoscape.

**Fig 12:**
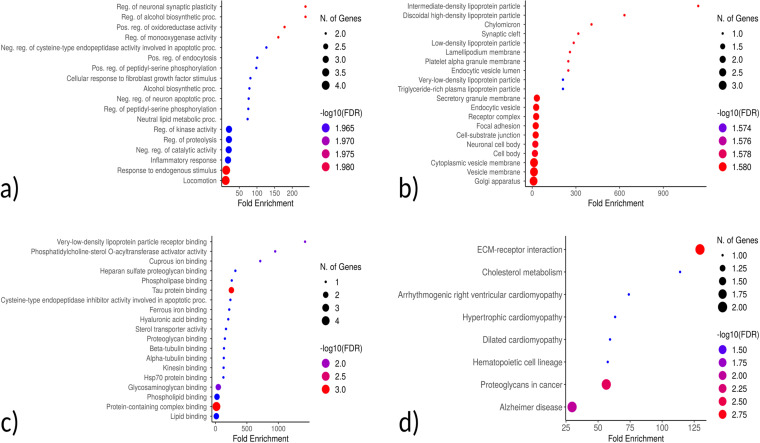
Enriched GO terms for the 4 identified hub genes. a) Biological Process b) Cellular Component c) Molecular Function d) KEGG Pathways.

The top 20 hub genes were calculated using the seven algorithms of the CytoHubba plugin in the Cytoscape software. After the intersection of the UpSet diagram was determined, four common hub genes were discovered: *APOE* (apolipoprotein E), *CD44* (*CD44* molecule), *SNCA* (synuclein alpha), and *ITGB5* (integrin subunit beta 5) (Fig 11). The mentioned hub genes were found to be enriched in several pathways (Fig 12) (Table 3)

**Table 3:** Expression profile of the identified hub genes. A minus sign (-) indicates downregulated genes.

Hub Gene	TSD (log2foldchange)	NPC (log2foldchange)	Description	Ensemble Gene ID
APOE	−2.46	−3.72	Apolipoprotein E	ENSG00000130203
ITGB5	−1.19	1.14	integrin subunit beta 5	ENSG00000082781
CD44	−1.28	2.76	CD44 molecule	ENSG00000026508
SNCA	−1.66	−1.01	synuclein alpha	ENSG00000145335

**Fig 13:**
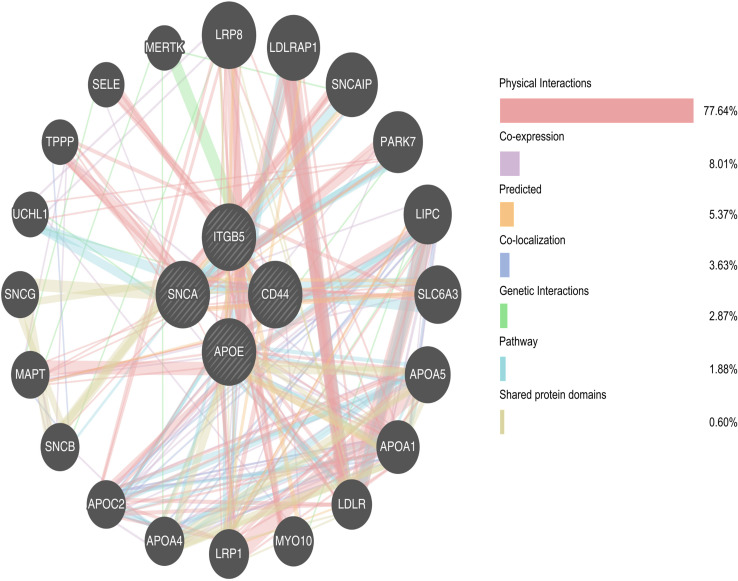
Gene-gene interaction network generated based on the four identified hub genes using the GeneMANIA platform http://genemania.org/. Nodes represent genes, with node size reflecting interaction degree, and edges represent functional associations (e.g., physical interactions, co-expression, pathway involvement). Edge colors indicate different types of interactions, as detailed in the legend. Central hub genes, including *APOE*, *CD44*, *SNCA*, and *ITGB5*, show high connectivity.

GO-BP showed that the genes were mainly involved in neuronal synaptic plasticity. KEGG pathway analysis revealed that the hub genes were primarily involved in ECM-receptor interaction and cholesterol metabolism, which aligns with TSD and NPC as lysosomal storage disorders that exhibit disrupted cellular adhesion and lipid homeostasis. These processes are critical for maintaining neuronal integrity and function, and their dysregulation is a hallmark of neurodegenerative diseases. The findings suggest that alterations in ECM-receptor interactions and cholesterol metabolism may underlie the shared neurodegenerative mechanisms of TSD and NPC, which contribute to progressive neuronal damage and loss observed in these disorders. The GeneMANIA platform was also utilized to construct a co-expression network of the hub genes (Fig 13) [25].

## Discussion

Our differential gene expression analysis aimed to identify pathways, mechanisms, and hub genes shared between TSD and NPC. The GO over-representation analysis of the common DEG dataset revealed that, in terms of biological processes, these genes were predominantly enriched in integrin-mediated cell adhesion. This pathway is a key contributor to neurological disorders, potentially causing imbalanced synaptic function in AD, intellectual disability, autism spectrum disorder, epilepsy, schizophrenia, addiction, and depression [26–28].

The KEGG pathway analysis of the common DEGs resulted in the identification of the ECM-receptor interaction pathway, which, along with the identification of *ITGB5* as a hub gene, further demonstrated the involvement of the DEGs in neurodegenerative phenotypes that can be seen in both TSD and NPC [29, 30]. ECM-integrin interaction regulates neurodevelopmental programs and is crucial in neuronal viability, function, differentiation, and plasticity [31]. It has also been reported that integrin signaling and focal adhesions are dysregulated in neurodegenerative diseases [32, 33]. Interactions of ECM with immunomodulatory factors such as *CD44* may speculate the consistent, respectively higher and lower expression of *ITGB5* and *CD44* genes in NPC and TSD patients [34]. *ITGB5* and *CD44* are both present in the ECM-receptor interaction KEGG pathway. KEGG pathway analysis showed phenylalanine metabolism as the most enriched pathway in the first 5-gene cluster identified by MCODE (Fig 9). Analysis of a Moroccan Jewish TSD patient had revealed an in-frame deletion (delta F) of one of the two adjacent phenylalanine codons that are present at positions 304 and 305 in the alpha-subunit sequence. The mutation impairs the subunit assembly of beta-HEXA, resulting in an absence of enzyme activity [35]. In another study on NPC mouse models, phenylalanine was found to be significantly upregulated in mutant mouse models compared to controls, which might serve as a biomarker for NPC. The increase in phenylalanine was likely associated with disrupted amino acid metabolism in the liver, which is a hallmark of metabolic dysfunction in NPC [36]. Phenylalanine is a precursor for tyrosine that contributes to the biosynthesis of catecholamines and other important molecules, which might be the reason why tyrosine metabolism is also seen as one of the enriched KEGG pathways.

KEGG and GO analyses were performed on the combined clusters, which revealed retinol metabolism as the most significantly enriched KEGG pathway (Fig 10). As we know, TSD patients are presented with cherry-red spots in their eyes, which is caused by an accumulation of gangliosides in the retinal cells, particularly in the macula [37]. Retinol metabolism plays a critical role in maintaining the visual cycle and proper functioning of the retina. Disruptions in this pathway, as suggested by the enrichment analysis, may contribute to the pathophysiological changes observed in TSD, including the characteristic cherry-red spots. Additionally, retinal degeneration is also present in NPC patients [38, 39]. A study was done on NPC patients, by which retinal degeneration was identified in NPC patients using optical coherence tomography (OCT) imaging, with a significant correlation observed between retinal neuroaxonal degeneration and clinical measurements. Their findings suggested that OCT imaging could be a valuable marker for assessing neurodegeneration in NPC following the onset of clinical symptoms [40].

AD, NPC, HD, and PD are among the neurodegenerative disorders that have been linked to dysregulation of cholesterol metabolism in the brain. There is a correlation between the development of AD and the expression of the genes that are involved in cholesterol biosynthesis (DHCR24, 24-dehydrocholesterol reductase) and cholesterol efflux (*APOE*, ATP-binding cassete transporter) [41]. It was found that some of the mutations in the LSDs patients were associated with neurodegenerative disorders as well as PD [42, 43]. In this study, KEGG pathway enrichment analysis of the identified hub genes revealed the Cholesterol Metabolism and AD pathways. These findings further support the shared mechanisms and neurological features linking TSD and NPC with other neurodegenerative diseases [44–47].

Hub gene analysis identified four important genes in the common DEG dataset: *APOE, CD44, SNCA, and ITGB5*. Many studies have provided strong evidence for the involvement of the *APOE* gene in neurodegeneration; *APOE* remains the most associated gene, impacting more than half of all AD cases. It is highly expressed in the brain and has a crucial role in cholesterol metabolism and transcription of amyloid precursor protein (APP) [48]. More specifically, the main function of *APOE* is to mediate lipid transportation in the brain and periphery [49]. Herein, it was found that the *APP* expression was significantly downregulated in the TSD dataset (LFC of -0.90), while it was upregulated in the NPC dataset (LFC of 0.54). The specific role of *APOE* in NPC pathogenesis is still unclear; however, its overexpression may be a compensatory role in eliminating accumulated lipids. The *APOE* expression was downregulated in both TSD and NPC. A recent study using human-induced pluripotent stem cell (iPSC) -derived cerebral organoids has shown that *APOE* deficiency impacts brain lipid homeostasis by modulating multiple cellular and molecular pathways. This deficiency can alter neural differentiation and cholesterol metabolism, potentially affecting overall brain function [50]. Research on NPC has shown similarities with AD, including neurofibrillary tangles. Mattsson et al. provided the first in vivo evidence for the effect of neuronal lipid accumulation that we have in NPC on gamma-secretase-dependent amyloid beta production, which has an important role in AD progression [51]. Another study found significant associations between *APOE* polymorphisms and NPC phenotypic severity, which supports the evidence for the role of *APOE* in NPC neuropathology. More specifically, alleles _APOE_4 and _APOE_2 were associated with increased and decreased severity of NPC, respectively [52]. Maulik et al. revealed that overexpression of APP in NPC1-deficient mice can negatively impact longevity and influence a wide spectrum of behavioral and neurological abnormalities [53,54]. Besides the significant role of *APOE* in AD progression, Harold et al. identified several other genes to be significantly associated with AD, including *CLU*, *PICALM,* and *BIN1* [55]. The *CLU* gene encodes clusterin, also known as apolipoprotein J, a brain-expressed lipoprotein involved in cholesterol metabolism, similar to *APOE*. Meanwhile, *PICALM* and *BIN1* encode proteins that play roles in clathrin-mediated internalization and endocytic recycling, respectively [56]. In NPC, we found *PICALM* to be slightly upregulated (0.25 LFC), *BIN1* expression was more than doubled (1.12 LFC), and *CLU* was moderately upregulated (0.50 LFC). On the other hand, in TSD, *PICALM* was slightly downregulated (-0.13 LFC), *BIN1* was also slightly downregulated (-0.33 LFC), and *CLU* was greatly downregulated (-2.00 LFC). Harold et al. proposed that alterations in *PICALM* function driven by genetic factors may disrupt synaptic activity, potentially by affecting synaptic vesicle cycling, which could elevate the risk of AD. Another possibility is that *PICALM* impacts AD risk by modulating APP processing through endocytic pathways, leading to changes in amyloid-beta levels.

*CD44* is a surface antigen that is expressed in numerous tissues, including immune and central nervous system cells. The *CD44* gene is involved in ECM organization, degradation, and cell adhesion. *CD44* was found to be strongly expressed in non-myelinating Schwann cells at the neuromuscular junction, and its expression increases during neurodegeneration-induced glial plasticity. *CD44* is enrolled in various functions in the nervous system, including axon guidance, synaptic transmission, and brain tumor development. *CD44* is mainly involved in the modulation of inflammation and therefore its overexpression in microglia can indicate the neuroinflammation pathogenesis in multiple sclerosis, PD and AD [57, 58]. Previous studies have demonstrated that *CD44* deficiency represses neuroinflammation and rescues dopaminergic neurons in a mouse model of PD [58]. Evidence for *CD44* overexpression was also found in AD patients who were carrying *CD44* splice variants [59]. Overexpression of *CD44* in the NPC data group (Table 2) may demonstrate the same neuroinflammation mechanism in progressive neurodegenrative diseases. Downregulation of *CD44* in TSD datasets which is in contrary of previous report in the spinal cord of mouse model of Sandhoff disease is revealing different mechanism of inflammation and neuronal loss [60]. Downregulation of *CD44* can lead to reduced tumor progression, impact cancer stem cell properties, alter critical cell signaling pathways [61]. However, during early developmental stages, *CD44* plays a key role in cell proliferation and growth. This might suggest that the TSD fetal samples were undergoing a developmental stage in which *CD44* expression was downregulated. Further experimental validation and studies on non-fetal patients would be necessary to confirm this.

The *SNCA* gene is mostly expressed in the brain, and it has been frequently shown that it is strongly involved in neurodegenerative diseases such as PD, AD, and Lewy bodies (LB) [62]. The product of the *SNCA* gene is the alpha-synuclein protein, and the abnormal accumulation of the alpha-synuclein protein in the brain can cause a group of neurodegenerative disorders called alpha-synucleinopathy. It has been found that *NPC1* gene variants might represent a risk or susceptibility factor in the development of alpha-synucleinopathies such as multiple system atrophy [63]. The *SNCA* gene is also widely recognized as the primary causal factor in the early development of familial PD [64], and its involvement in PD is strongly supported by the facts that aggregated *SNCA* is the primary component of LB in sporadic PD, and missense and copy number variations in the *SNCA* gene are known to induce hereditary PD [65]. In our analysis, *SNCA* was downregulated in both TSD and NPC. The *SNCA* gene has been shown to be downregulated in AD as well [66], which aligns with our finding in both NPC and TSD datasets.

The *ITGB5* gene encoding *integrin* *β5* has been frequently found to be overexpressed in the progression and invasion of various types of human cancers [67–69]. Additionally, *ITGB5* expression has been associated with neurodegenerative diseases such as PD, HD, and AD as well [70]. The T al. result implies an association of *ITGB5* with amyloid accumulation and brain atrophy, as it was associated with slower atrophy in the hippocampus, ventricle, and entorhinal cortex but faster atrophy in the parietal gray matter [71]. In addition, they concluded that higher levels of *ITGB5* may act as a marker of reduced dementia risk, as a higher expression level of *ITGB5* is associated with reduced odds of cognitive impairment. Interestingly, overexpression of *ITGB5* was found in amyotrophic lateral sclerosis, which is a progressive neurodegenerative disease, consistent with the progressive pattern of NPC [72]. It suggests that *ITGB5* expression has the potential to serve as a novel biomarker for differentiating early and late stages of chronic neurodegenerative diseases, age-related memory deficits, and AD [32]. The contrasting expression patterns of *ITGB5* between NPC and TSD (upregulated in NPC and downregulated in TSD) may suggest a differential involvement in the cellular mechanisms of these disorders. Specifically, the upregulation of *ITGB5* in NPC could indicate its role in pathways contributing to progressive neurodegeneration, such as enhanced ECM-receptor interactions or altered cell adhesion, which are hallmarks of NPC pathology.

In terms of cellular components, the identified hub genes were mostly related to mast cell granules (known as secretory lysosomes), which contain both lysosomal proteins such as acid hydrolases, e.g., β-hexosaminidase, as well as mediators such as histamine, and can secrete both together. As previously shown, the involvement of fascia adherence indicates cardiovascular interventions. A recently published study has shown that certain indel mutations in the *NPC1* gene responsible for NPC increase the risk of cardiac sudden death [73]. It has also been shown that *NPC1* plays a critical role in atherosclerotic progression [74].

In this study, we showed that there are shared pathways, mechanisms, and hub genes between TSD and NPC, highlighting integrin-mediated cell adhesion and ECM-receptor interaction as central pathways associated with neurodegenerative phenotypes. Through hub gene analysis, we identified *APOE*, *CD44*, *SNCA*, and *ITGB5* as key genes, demonstrating their potential roles in neurodegeneration through dysregulated cholesterol metabolism, neuroinflammation, and alpha-synuclein aggregation. These findings strengthen the link between lysosomal storage disorders and broader neurodegenerative processes, providing insights into shared pathophysiological mechanisms.

Despite these advances, this study has several limitations. First, the analysis relies on publicly available datasets, which may introduce biases due to variations in data quality, experimental conditions, and sample heterogeneity. Second, our study is primarily computational, lacking direct experimental validation of the identified hub genes and pathways. Lastly, the role of identified genes, particularly their contrasting expression patterns in TSD and NPC, remains speculative without further mechanistic studies. It is also notable that the TSD dataset focused on the pre-symptomatic stage of TSD, whereas the NPC organoids may represent more advanced phenotypes of the disease due to the accumulation of cholesterol and lysosomal dysfunction that was observed.

To validate the hub genes and their roles, several experimental approaches could be employed. For instance, quantitative PCR (qPCR) and Western blot analyses can confirm the expression levels of *APOE*, *CD44*, *SNCA*, and *ITGB5* in patient-derived cells or relevant model systems. Immunohistochemistry (IHC) could localize protein expression in affected tissues, while RNA interference (RNAi) or CRISPR-Cas9 gene editing can assess the functional consequences of gene knockdown or overexpression. Additionally, co-immunoprecipitation and mass spectrometry could identify interacting partners and pathways involving these genes. These methods would provide robust experimental evidence to support our computational findings and elucidate their precise roles in TSD and NPC pathogenesis.

## Conclusion

In conclusion, our analysis revealed shared molecular mechanisms between TSD and NPC and common neurodegenerative diseases such as AD and PD. Integrin-mediated cell adhesion, ECM-receptor interaction, and dysregulation of cholesterol metabolism emerge as key pathways. Hub genes *APOE*, *CD44*, *SNCA*, and *ITGB5* exhibit distinct expression patterns, linking them to neurodegenerative phenotypes. Notably, the dysregulation of cholesterol metabolism, particularly the significant role of APOE, further emphasizes the connection between LSDs and neurodegenerative disorders. Opposite expression patterns of the hub genes, except for *SNCA,* demand for functional studies to shed light on how GM2 ganglioside accumulation differentially affects cellular responses compared to sphingomyelin and cholesterol aggregation while leading to the same outcome, neurodegeneration. Further investigations are being conducted in ongoing research analyzing the expression of hub genes in patients with neurodegenerative diseases to be validated as early biomarkers.
